# Supplementary material for: Hydroxychloroquine-mediated inhibition of SARS-CoV-2 entry is attenuated by TMPRSS2
Source: PLoS Pathog. 2021 Jan 19;17(1):e1009212. doi: 10.1371/journal.ppat.1009212 (PMC7845965; doi:10.1371/journal.ppat.1009212)
Supplement: Attachment — Submitted filename: Ou et al.docx [file ppat.1009212.s002.docx]

PPATHOGENS-D-20-01563

The S proteins of SARS-CoV-1 and -CoV-2 are dependent on proteolytic cleave, either by TMPRSS2 at the plasma membrane or by cathepsin L in low pH endosomes, before they can mediate fusion with the target cell membrane and viral entry. Using cathepsin inhibitors, cell lines differing in TMPRSS2 expression, the TMPRSS2 inhibitor camostat, and varying concentrations of hydroxychloroquine, Ou et al. show that SARS-CoV-2 is more dependent on TMPRSS2 for S protein cleavage and entry than SARS-CoV-1. As a consequence, SARS-CoV-2 is more resistant than SARS-CoV-1 to the effects of hydroxychloroquine, which inhibits endosomal acidification and low pH cathepsin-mediated activation of the S protein. The dependence of SARS-CoV-2 on TMPRSS2 also correlates with the presence of a polybasic cleavage site in S for furin-mediated cleavage in the producer cell.

The data showing that SARS-CoV-2 is more dependent on TMPRSS2 than SARS-CoV-1, and is therefore less sensitive to hydrochoroquine, is convincing and has important clinical implications, since it helps to explain why hydrochoroquine was able to inhibit SARS2 infectivity in Vero E6 lacking TMPRSS2, but ultimately failed to confer a beneficial effect in clinical trials. However, the relationship between furin-mediated cleavage of the SARS2 S protein and greater dependence of TMPRSS2 is less complete, since it only compares the infectivity of wild-type, furin-site knockout (FKO) and D614G viruses. Ideally, one would like to see biochemical confirmation of the effects of these mutations on S protein sensitivity to furin and/or an increase in TMPRSS2 utilization by the SARS1 S protein after the introduction of a polybasic furin cleavage site.

Major comments:

1. Differences in high versus low TMPRSS2 expression in stable 293T-ACE2 cell lines (Fig. 3) need to be supported by flow cytometry and/or western blot data comparing differences in expression.
2. The effects of mutations in the polybasic cleavage on sensitive to furin (Fig. 5) should be supported by western blots.
3. Does the introduction of the furin cleavage site into the SARS-CoV-1 S protein increase its ability to use TMPRSS2?

Minor comments:

1. The manuscript would benefit from careful editing to correct numerous instances of misspelled or missing words (e.g. “facitiatea”; “hydroxychloroquine alone inhibited SARS1-PV more than camostat”).
2. Statements in the Introduction referring to hydroxychloroquine clinical trials and the EC50 of hydroxychloroquine in Vero E6 are missing references. This is also true of the statement that D614G partially restores S protein stability in the Results.
3. In the legend for Figure 5, the first line should refer to Fig. 2A (not Fig. 1A).
