# Supplementary material for: Hydroxychloroquine-mediated inhibition of SARS-CoV-2 entry is attenuated by TMPRSS2
Source: PLoS Pathog. 2021 Jan 19;17(1):e1009212. doi: 10.1371/journal.ppat.1009212 (PMC7845965; doi:10.1371/journal.ppat.1009212)
Supplement: Attachment — Submitted filename: Response_to_Reviewers.doc [file ppat.1009212.s003.doc]

**RESPONSES TO REVIEWERS, Ou et al.**

**Editors’ comments:**

Dear Dr. Farzan,

…

I had invited 3 experts who agreed to review this manuscript. This pandemic has made it difficult to secure the last review in a timely fashion, so only two reviews are attached.  Both reviewers recognized the significance of the topic, but also that the rigor was lacking to some extent. This must be addressed (number of repeats, statistics, etc.). Please try to meet their major concerns more than half-way. I would add that since the study hinges quite a bit on proteolytic activation of SARS-CoV-2 entry, biochemical support for how HCQ modulates TMPRSS2-dependent vs independent cleavage and entry should be provided. This would greatly enhance the impact of your paper, as the field is now mature enough to link proteolytic activation with biochemical evidence of cognate cleavage.

We cannot make any decision about publication until we have seen the revised manuscript and your response to the reviewers' comments. Depending on how thorough you address the reviewer's comments, your revised manuscript may be sent to reviewers for further evaluation. We try to avoid multiple rounds of review, so I urge you to provide a well-considered revision that will enable me to render a decision without the further delay of re-review.

…

Thank you for this consideration. As mentioned above and described in detail below, we will be able to address all of the reviewers’ concerns except for their request to repeat experiments with infectious virus.

We have also provided additional biochemical support for our main conclusion, verifying the expected S1/S2 cleavage by western blot and most notably by introducing a furin-cleavage site into the SARS-CoV-1 S protein and demonstrating that the resulting S protein is more dependent on TMPRSS2.

**Reviewers’ comments:**

Reviewer #1:

In the manuscript by Ou et al, the authors assess the ability of hydroxychloroquine to inhibit entry of SARS-CoV-2. They demonstrate that hydroxychloroquine has limited activity against TMPRSS2-mediated entry but is effective against Cathepsin L-mediated entry. They further demonstrate that hydroxychloroquine is synergistic with the TMPRSS2 inhibitor Camostat. Unraveling the mechanism and potential utility (or lack thereof) of entry inhibitors such as hydroxychloroquine is of significant interest for the treatment of COVID-19. However, enthusiasm for the manuscript is tempered by the complete reliance on pseudovirus assays and transgenic cell lines and questionable experimental rigor (as it was poorly described)

1) The entire manuscript depends on pseudovirus. While a powerful system, it may not reflect the stoichiometry, conformation, or ratio of cleaved:uncleaved spike on native SARS-CoV-2 virions. Validation of key findings herein with native SARS-CoV-2 virions (WT, D614G, and furin mutant) is critical. The WT and D614G viruses are readily available and the experiments are straightforward with the reagents in hand.

As described in the cover letter, although we have directly compared WT, D614G, and furin-mutant pseudoviruses, we have not done so with infectious virus. We would note that several studies now demonstrate tight correlations between pseudovirus and plaque assays with infectious virus (e.g. Liu et al., *Nature* 2020; Folegatti et al., *Lancet* 2020), and that the infectious virus assay requested has already been performed in Hoffman et al. (*Nature*, 2020; 585:588-590). Note that we have extended Hoffman et al.’s results in two ways. First, we have shown that SARS-CoV-1 and SARS-CoV-2 are differentially dependent on TMPRSS2 and cathepsin L, and differentially sensitive to inhibitors of these processes. Second, we have shown that this difference is due to the presence or absence of a furin site.

2) The entire manuscript exclusively utilizes 293T cells overexpressing key entry molecules ACE2 and TMPRSS2. Key experiments should be reproduced in a physiologically relevant system such as a primary human airway culture system (or ACE2 transgenic mice).

We agree with the reviewer’s concern on physiological relevance using only 293T cells. We have reproduced the viral entry inhibition assays on multiple cells including human kidney epithelial cells (Vero) and human lung epithelial cells (H1299, H1975, and Calu-3), each of which confirmed our findings on 293T cells with TMPRSS2 overexpression.

3) Each figure legend should state how many independent experiments were performed and with how many replicates per experiment. It appears that some figures represent only a single experiment with multiple technical replicates. If true, this would be unacceptable for publication.

Our apologies for the oversight. All the viral entry inhibition assays were performed with triplicate samples for three times. The other experiments (flow cytometry for example) were performed with duplicates for at least twice. The data we show are one representative of several independent experiments (such as in Fig 2A) or the mean value calculated from multiple experiments (such as in Fig 2B). We have now indicated the number of replicates and independent experiments in all figures.

Reviewer #1: Minor

4) Fig 2A, the authors should show a mock virus to reveal the limit of detection of the assay.

We have now included VSV-G pseudoviruses in Fig 2 A to address this issue.

5) The figure 2 title says “inhibiting endosomal proteases does not reduce SARS2 infectivity in TMPRSS2 cells”. The E64D and ZIIIFMK data refute this as all 10uM data points are below the 100% line. This reviewer appreciates that the magnitude of reduction is very modest (and arguably not biologically meaningful) but the figure title as written not accurate given the data.

We agreed with the reviewer’s comment. To be more precise, we have now corrected this title to “SARS-CoV-2 is resistant to endosomal protease inhibitors in cells over-expressing TMPRSS2”.

6) Histograms should show individual data points and not simply box and whiskers.

We have now provided individual data points to our figures (Fig 2B-C, Fig 3B, and Fig 5D).

7) Last line of intro should be changed to “may reflect” as the failure of hydroxychloroquine in vivo is unclear.

The reviewer is correct, and we have changed the text accordingly.

8) Needs careful proofreading as there are numerous typos

Our apologies. We have tried to catch all typos and improve the text.

Reviewer #2:

“The S proteins of SARS-CoV-1 and -CoV-2 are dependent on proteolytic cleave, either by TMPRSS2 at the plasma membrane or by cathepsin L in low pH endosomes, before they can mediate fusion with the target cell membrane and viral entry. Using cathepsin inhibitors, cell lines differing in TMPRSS2 expression, the TMPRSS2 inhibitor camostat, and varying concentrations of hydroxychloroquine, Ou et al. show that SARS-CoV-2 is more dependent on TMPRSS2 for S protein cleavage and entry than SARS-CoV-1. As a consequence, SARS-CoV-2 is more resistant than SARS-CoV-1 to the effects of hydroxychloroquine, which inhibits endosomal acidification and low pH cathepsin-mediated activation of the S protein. The dependence of SARS-CoV-2 on TMPRSS2 also correlates with the presence of a polybasic cleavage site in S for furin-mediated cleavage in the producer cell.

The data showing that SARS-CoV-2 is more dependent on TMPRSS2 than SARS-CoV-1, and is therefore less sensitive to hydrochoroquine, is convincing and has important clinical implications, since it helps to explain why hydrochoroquine was able to inhibit SARS2 infectivity in Vero E6 lacking TMPRSS2, but ultimately failed to confer a beneficial effect in clinical trials.

However, the relationship between furin-mediated cleavage of the SARS2 S protein and greater dependence of TMPRSS2 is less complete, since it only compares the infectivity of wild-type, furin-site knockout (FKO) and D614G viruses. Ideally, one would like to see biochemical confirmation of the effects of these mutations on S protein sensitivity to furin and/or an increase in TMPRSS2 utilization by the SARS1 S protein after the introduction of a polybasic furin cleavage site.”

As described below, we have now added data showing studies of an engineered SARS-CoV-1 S protein in which a furin site has been introduced. These studies provide strong additional support our assertion that TMPRSS2 utilization is dependent on furin-mediate cleavage in the target cells.

Major comments:

1. Differences in high versus low TMPRSS2 expression in stable 293T-ACE2 cell lines (Fig. 3) need to be supported by flow cytometry and/or western blot data comparing differences in expression.

To address this concern, we have included cell-surface staining data (Fig 2A) to validate the transient and stable overexpression of TMPRSS2 in 293T-ACE2 cell lines. In addition, we included new data with other cell types (H1299, H1975, Calu-3 and Vero). Their TMPRSS2 expression levels are shown by western blots.

2. The effects of mutations in the polybasic cleavage on sensitive to furin (Fig. 5) should be supported by western blots.

We have now included western blot data showing that these mutations (D614G and furin-site knockout) do indeed alter cleavage of SARS-CoV-2 spike proteins at the S1/S2 boundary. These observations were in agreement with another recent publication from our groups (Zhang et al., 2020; *Nature Communications*, in press).

3. Does the introduction of the furin cleavage site into the SARS-CoV-1 S protein increase its ability to use TMPRSS2?

We have introduced this furin site into the SARS-1CoV-1 S-protein, shown by Western blot that this furin site is functional, and characterized its infectivity and sensitively to different entry-inhibiting compounds (hydroxychloroquine and camostat), compared to the wildtype SARS-CoV-1. We observed that the addition of furin-cleavage site increased the dependence of SARS-CoV-1 on TMPRSS2 for viral entry, consistent with our hypothesis.

Minor comments:

1. The manuscript would benefit from careful editing to correct numerous instances of misspelled or missing words (e.g. “facitiatea”; “hydroxychloroquine alone inhibited SARS1-PV more than camostat”).

We have done our best to proofread the manuscript more carefully.

2. Statements in the Introduction referring to hydroxychloroquine clinical trials and the EC50 of hydroxychloroquine in Vero E6 are missing references. This is also true of the statement that D614G partially restores S protein stability in the Results.

We have added the appropriate references.

3. In the legend for Figure 5, the first line should refer to Fig. 2A (not Fig. 1A).

This mistake has been corrected.
